# Language and Short-Term Memory: The Role of Perceptual-Motor Affordance

**DOI:** 10.1037/a0036845

**Published:** 2014-05-05

**Authors:** Bill Macken, John C. Taylor, Dylan M. Jones

**Affiliations:** 1 School of Psychology, Cardiff University

**Keywords:** lexicality effect, redintegration, auditory perception, articulatory fluency, modality

## Abstract

The advantage for real words over nonwords in serial recall—the *lexicality effect*—is typically attributed to support for item-level phonology, either via redintegration, whereby partially degraded short-term traces are “cleaned up” via support from long-term representations of the phonological material or via the more robust temporary activation of long-term lexical phonological knowledge that derives from its combination with established lexical and semantic levels of representation. The much smaller effect of lexicality in serial recognition, where the items are re-presented in the recognition cue, is attributed either to the minimal role for redintegration from long-term memory or to the minimal role for item memory itself in such retrieval conditions. We show that the reduced lexicality effect in serial recognition is not a function of the retrieval conditions, but rather because previous demonstrations have used auditory presentation, and we demonstrate a robust lexicality effect for visual serial recognition in a setting where auditory presentation produces no such effect. Furthermore, this effect is abolished under conditions of articulatory suppression. We argue that linguistic knowledge affects the readiness with which verbal material is segmentally recoded via speech motor processes that support rehearsal and therefore affects tasks that involve recoding. On the other hand, auditory perceptual organization affords sequence matching in the absence of such a requirement for segmental recoding and therefore does not show such effects of linguistic knowledge.

Since its inception within cognitive psychology, short-term memory has typically been cast as a mode of processing both distinct from and interactive with long-term memory. There are accounts that characterize this distinction either in terms of separate systems and processes for short- versus long-term retention (e.g., Baddeley, 2012; Baddeley, Gathercole, & Papagno, 1998) or in terms of short-term memory as an activated state of long-term representations (e.g., Cowan, 1995; Crowder, 1993; MacDonald & Christiansen, 2002; Martin & Saffran, 1997; Ruchkin, Grafman, Cameron, & Berndt, 2004). From both perspectives, the interaction between short- and long-term memory involves both the transmission of new information into long-term storage and reciprocal input from long-term knowledge to support short-term information processing. A key empirical hallmark of the latter—and the focus of our current concerns—is the superior memory typically observed for phonological material that closely corresponds to the rememberer’s long-term linguistic knowledge relative to material that deviates from it. So, short-term memory for sequences of words is typically superior to that for sequences of nonwords—the so-called *lexicality* effect^1^ (e.g., Gathercole, Pickering, Hall, & Peaker, 2001; Hulme, Maughan, & Brown, 1991; Roodenrys, Hulme, & Brown, 1993)

Critical to theoretical accounts of this lexicality effect is that its presence varies depending on retrieval conditions: it appears robustly and reliably in serial *recall*, but when memory is tested via serial *recognition,* the effect is attenuated to varying degrees compared with that found in recall (Gathercole et al., 2001; Jefferies, Frankish, & Lambon-Ralph, 2006; see also Thorn, Gathercole, & Frankish, 2002, for a similar finding in relation to first- vs, second-language material in bilinguals). The key functional distinction here between recall and recognition is that, in the former, an original sequence is presented and must subsequently be reproduced in some form (spoken, written, typed, and so on) while in the latter, a standard sequence is presented, followed by the presentation of a second, test, sequence that is either identical or subtly different from it (e.g., by transposition of two adjacent items), and the task is to judge whether the two sequences are the same or different. Critically, therefore, serial recall typically involves a setting in which the participant must reproduce the items from the original sequence in their original order, while serial recognition involves the participant being presented again with those original items and being required to judge whether those items are in the same order as their original presentation.

The reduced effect of long-term lexical knowledge when short-term memory is tested via serial recognition rather than recall has been used to argue the case for short-term memory as a distinct mode of representation in itself, rather than it being an activated portion of long-term storage (e.g., Baddeley, 2003). This interaction of lexical status and retrieval condition plays a key role in such accounts of the impact of long-term memory on short-term performance in the following way: Phonological material encoded into short-term storage is subject to degradation (due to decay or interference) such that information about item identity is lost over time. The existence of permanent linguistic (lexical–phonological) representations in long-term memory that correspond to those degraded short-term representations means that a match is available that can be used to support the recovery of the identity of those volatile short-term codes—a process referred to as *redintegration* (e.g., Hulme et al., 1993; Roodenrys et al., 1993; Schweikert, 1993). Therefore, in serial recall, where the task is to reproduce an intact sequence from the degraded information in short-term memory, the greater the availability and accessibility of corresponding long-term representations, the more successful recall performance will be. However, in the case of serial recognition, where all the items are re-presented in the test sequence, the question of their availability and accessibility in long-term storage is obviated; the critical items themselves are present in the recognition cue, and so the cue itself provides the match against which the degraded short-term representations may be compared. In this way, serial recognition performance is less affected, compared with serial recall, by effects arising from item redintegration.

That the effect of long-term linguistic knowledge in short-term memory varies depending on the precise nature of the task appears to undermine the view that short-term memory is not separate from, but rather the activated portion of, long-term memory, and indeed, the deployment of a redintegrative process has proved very successful in modeling the various manifestations of long-term representations in short-term memory performance (e.g., Burgess & Hitch, 1999; Henson, 1998; Hulme et al., 1997; Hulme, Newton, Cowan, Stuart, & Brown, 1999; Lewandowsky, 2000; Nairne, 1990; Page & Norris, 1998). Therefore, the interaction between lexicality and retrieval condition plays a key role in sustaining this particular account of the distinct and interactive status of short- and long-term memory systems and codes.

Another account of the role of long-term linguistic knowledge in short-term memory performance makes no such distinction between long- and short-term phonological information but rather identifies short-term memory performance as being based on the temporary activation of the same verbal codes that underpin linguistic processing more generally (e.g., MacDonald & Christiansen, 2002; Martin & Saffran, 1997; Ruchkin, Grafman, Cameron, & Berndt, 2004). From this perspective, the advantage accruing to lexically familiar material in short-term memory tasks derives from the sustained activation of phonological information due to interactive activation deriving from mutual connections between lexical and semantic levels of representation for words and their corresponding phonological features (e.g., Dell & O’Seadhgha, 1992; Martin & Gupta, 2004). By the same token, since the phonological features of lexical representations benefit from repeated co-activation whenever the corresponding lexical representation is activated within the language system, the phonological constituents of lexical material are less likely to be subject to sublexical errors in recall (e.g., Jefferies et al., 2006; Jefferies, Frankish, & Noble, 2009; Patterson, Graham, & Hodge, 1994). From this perspective, linguistic knowledge is expected to have a variety of effects—deriving from semantic, lexical, and phonological levels of representation—that should be evident in various aspects and stages of short-term memory task performance (see e.g., Allen & Hulme, 2006; Thorn, Gathercole, & Frankish, 2002; Ruchkin et al., 2004).

On the face of it, such an account would appear to predict effects of lexicality more generally in short-term memory performance (and indeed, there are neurophysiological effects of lexicality across a variety of task formats and stages: Ruchkin et al., 2004), while the evidence, as we have discussed, suggests that its effects are dependent on retrieval conditions. However, an important aspect of the role of linguistic factors in this respect is that they appear to be especially important for the retention of item information in short-term memory, as opposed to order information (e.g., Gathercole et al., 2001; Hulme et al., 1997). Therefore, serial recognition, in re-presenting all item information at retrieval, is likely to be a setting in which the influence of such factors is attenuated, not because it provides copies of the target items against which degraded short-term codes may be compared but because the task reduces the burden on item memory—and therefore is less sensitive to effects that arise in that aspect of maintenance—and instead only requires a judgment about order across the two sequences. The finding that a nominally more sensitive serial recognition task that requires the detection of changes in the order of phonemes across words exhibits substantial effects of lexicality, while the standard version requiring the detection of changes in word or syllable order is less affected, lends support to this sensitivity-based interpretation of the interaction between lexicality and retrieval condition (Jefferies et al., 2006). We return to this particular finding momentarily.

The two accounts of the lexicality effect we have outlined, while differing in the conceptualization of the precise nature of the phonological representations that underpin performance, nonetheless share a focus on factors affecting the processing of item-level phonological information. Our approach to the question of the lexicality effect in short-term memory differs from each of these and starts from the observation that in both the accounts we have described, there is what we argue to be a critical elision; namely, the role of the modality in which the to-be-remembered material is presented. Put simply, while serial recall is commonly implemented with both visual and auditory presentation (sometimes as a matter of interest, sometimes merely as procedural expedience), serial recognition has almost without exception employed auditory presentation. Given that theories of short-term memory almost universally invoke a core, abstract, memorial level of representation in relation to which both the perceptual processes that provide input to it and the motor processes that mediate output are seen as auxiliary mechanisms, such an oversight is perhaps not surprising and not usually considered critical. Indeed, while theories of short-term memory have to address the question of modality (since there are clear differences between, for example, auditory and visual serial recall), it is typically treated as a matter of differential means of access to the “core” phonological form, rather than as a question of fundamentally different representational forms per se (e.g., Burgess & Hitch, 1999; Henson, 1998; Page & Norris, 1998) However, because an increasing body of theoretical and empirical work successfully accounts for short-term memory phenomena in terms of the task-specific deployment of perceptual, motor, and linguistic (as opposed to specifically memory) processes, then the question of modality in this context comes into sharp relief (see e.g., Acheson & MacDonald, 2009; Gupta, 1996; Hanley & Hayes, 2012; Hickok & Poeppel, 2004; Hughes, Marsh, & Jones, 2009; Jones, Hughes, & Macken, 2006; Jones, Macken, & Nicholls, 2004; Jonides et al., 2008; Maidment & Macken, 2012; Maidment, Macken, & Jones, 2013; Postle, 2006; Wilson, 2001).

In light of the way in which the particular kinds of task requirements involved in serial recall and serial recognition map on to the types of sequence processing abilities that are afforded by auditory (as opposed to visual–verbal) presentation, the exclusive use of auditory presentation in serial recognition takes on major theoretical importance. The particular relevance here is that auditory presentation affords sequence-processing functions based on obligatory and purely perceptual mechanisms that are not available for sequential visual presentation and that are also dissociable from the type of segmental sequence processing accomplished via deliberate, subvocal, speech-motor-control processes that may be engaged regardless of modality of presentation (see, e.g., Burton, Small, & Blumstein, 2000; Jones et al., 2004; Macken, Tremblay, Houghton, Nicholls, & Jones 2003; Warren, 1999). Functional distinctions between auditory and visual serial recall have long been noted. Specifically, auditory presentation typically affords enhanced serial recall for material toward the end of a sequence relative to that afforded by visual presentation, and further, that enhanced performance is eliminated in the presence of a redundant auditory item occurring after the end of the list (Crowder & Morton 1969). Critically in the current context, these distinctions between sequence processing across modality reflect a contribution to performance of processes involved in the perceptual organization of acoustic sequences that is both obligatory and distinct from the type of deliberate subvocal processing that may also subsume sequence processing (Jones et al., 2004; Macken et al., 2003; Maidment & Macken, 2012).

The distinction between obligatory auditory sequence processing and deliberate subvocal processing associated with speech-motor-control mechanisms is evidenced in a variety of ways. So, for example, the ability to report the order of a series of unrelated sounds (e.g., a buzz, a hiss, a click, and a vowel sound) depends on the sounds being presented at a rate sufficiently slow as to allow time for verbal recoding of each sound. However, the ability to discriminate between different orderings of the sounds may be accomplished at rates of presentation too fast to allow for such recoding (e.g., Warren, Obusek, & Farmer, 1969). The important implication is that the global perceptual pattern of the sound sequence, not the individual identity of each item, is the basis for accomplishing such a matching task. This conclusion is further warranted by the finding that such discrimination ability is dissociated from the ability to identify which particular elements in the sequence are the source of the difference—an ability that again only emerges at rates of presentation sufficiently slow to allow for verbal recoding (see e.g., Warren, 1999).

This dissociation is also evident in neuroscientific evidence showing that judgments about auditory verbal stimuli that require some sort of segmental processing typically engage frontal cortical motor areas involved in speech production along with posterior auditory areas, while judgments about such stimuli that are based on global temporal acoustic properties merely engage posterior, perceptual-processing areas (e.g., Burton et al., 2000; Norris & Wise, 2000; Wise et al., 1991; Zatorre, Meyer, Gjedde, & Evans, 1996). That processes underpinning auditory sequence discrimination may be dissociated from processes involved in segmental subvocal recoding of those sequences is also clear from evidence showing that such sequence discrimination may be accomplished under conditions where such recoding is impeded by requiring participants to engage in concurrent articulatory suppression during the auditory sequence-matching task (Macken, Phelps, & Jones, 2009).

This understanding of the nature of auditory perceptual sequence processing points to an alternative account of the interaction between retrieval conditions and lexicality of the memory material that is couched neither in terms of the storage and retrieval of volatile short-term representations nor in terms of the temporary activation of long-term lexical–phonological knowledge, but rather in terms of the distinct and combined action of perceptual and motor processes in different types of short-term memory tasks. As noted, since serial recall requires reproduction of a presented sequence, it necessarily requires some sort of recoding of that original verbal sequence to enable its subsequent output,^2^ and therefore, the readiness and fluency with which such recoding may be carried out affects performance. Indeed, there is abundant evidence that familiarity with a set of verbal material (of which lexical status is an example) influences the fluency of articulatory processing afforded by that material. For example, the rate of articulation of high-frequency words is considerably faster than that for low-frequency words, an effect primarily evident when sequences of words, rather than single words in isolation, are to be produced (even when those word sequences themselves have been familiarized, e.g., Woodward, Macken, & Jones, 2008; Wright, 1979). The way in which lexical frequency impacts on processes associated with reduced articulatory complexity, or lenition, in connected speech provides a plausible basis for this increased fluency as a function of lexical frequency (see e.g., Bybee, 2010; Hooper, 1976). Critically in this respect, articulatory complexity, rather than duration per se, has been shown to determine short-term serial recall (e.g., Service, 1998), suggesting that the key issue is the facility with which the participant’s linguistic skill may be deployed to manipulate a segmental representation of the memory sequence. From this standpoint, the evidence of a substantial effect of lexicality on an auditory serial recognition task requiring judgments about phoneme—rather than word or syllable—order described previously (Jefferies et al., 2006) is less a manifestation of different sensitivity per se of the two task formats, but rather of the involvement of segmental, speech-motor processes in manipulating subsyllabic segments (e.g., Burton et al., 2000; Hickok & Poeppel, 2004).

The key conclusion we draw from this is that the impact of lexical status is especially evident in those settings where verbal material has to be segmentally encoded in some articulatory form. We propose, therefore, that it is not the different retrieval conditions that account for greater effects of lexical status in serial recall compared with those found with serial recognition. Rather, we argue, it is because serial recognition has been implemented auditorily and therefore has afforded global auditory pattern-matching without segmental recoding, while serial recall, regardless of modality of presentation, necessarily requires some such recoding and therefore, the readiness with which the material affords segmental coding has an impact on performance. Whether the interaction between lexicality and retrieval conditions reported elsewhere is due to the redundancy of redintegration in recognition or due to the reduced sensitivity of recognition to linguistic effects, presenting the standard and test sequences in the recognition task in a visual–verbal format should lead to broadly the same outcome, since, just as in the auditory case, all the item information is re-presented in the test sequence. On the other hand, if it is the affordances deriving from auditory perceptual processing within serial recognition that attenuate the lexicality effect in that setting, then visual serial recognition should be amenable to the effects of lexicality, as is serial recall. In the experiments that follow, we tested this possibility by manipulating both the retrieval conditions (i.e., recall vs. recognition) and the modality (visual vs. auditory) in which the sequences are presented.

## Experiment 1

For the sake of correspondence between our findings and already-existing demonstrations of an interaction between lexicality and retrieval conditions, we adopted methodological aspects of prior demonstrations of such effects from Gathercole et al. (2001). This involved using five-item sequences of both real words and nonwords conforming to a consonant (or consonant cluster)–vowel–consonant (or consonant cluster; CVC) sound structure. We used a faster rate of presentation than that used by Gathercole et al. with a view to optimizing the conditions under which auditory perceptual processing would manifest itself in performance. Specifically, since we are proposing that auditory serial recognition can be seen as a task involving, not the short-term maintenance of phonological information as such, but rather perceptual pattern-matching between two auditory “objects”; then utilizing task parameters likely to enhance that auditory object formation should increase the perceptual contribution to the task and, concomitantly, according to our argument, reduce the effect of lexicality. At the same time, since the visual serial recognition and both serial recall tasks do not afford this auditory pattern-matching, the same task parameters should reveal a lexicality effect in each case (see, e.g., Maidment & Macken, 2012; Warren, 1999). Therefore, the same stimuli and timings were used in both auditory and visual forms of the tasks; in Experiment 1A, we tested serial recall of sequences factorially combining lexicality and modality, while in Experiment 1B we tested performance with serial recognition.

### Experiment 1A

#### Method

##### Participants

Sixteen participants (13 women, mean age 21 years) were recruited from the Cardiff University Human Participant Panel. Informed consent was obtained in accordance with Cardiff University School of Psychology ethics procedures.

##### Materials

Verbal stimuli took the form of single-syllable words and pronounceable nonwords, all with CVC sound patterns (125 of each), drawn from those used by Gathercole et al. (2001). Two sets (one of words and one of nonwords) of 25 unique five-item sequences were constructed without repetition of any item. Each participant was presented with 20 different randomly sampled sequences from each of these sets. One set consisted exclusively of words, the other exclusively of nonwords. Sequences were constructed such that no two items in a sequence shared the same vowel segment, and there was no more than one common consonant segment across items within a sequence.

Visual stimuli were presented in 40-point, Arial font (white text on a black background) at a resolution of 72 dpi. Auditory stimuli were recorded in a monotone male voice at a sample rate 44.1 kHz/16-bit using a condenser microphone and digitized using Audacity 1.3 software, on an Apple Mac running OS 10.6 (Apple Corp., Cupertino, CA). Individual items were then edited to a duration of 250 ms and to an amplitude of 70 dB. In order to minimize pitch discontinuities at the boundaries of successive items within a sequence, *F*_0_ of each item was normalized to 125 Hz (the median pitch of all 250 items) using Praat software (Boersma, 2001).

Experiments were undertaken in a soundproof booth. Stimuli were presented either visually via a computer screen or auditorily via headphones. Spoken recall responses for each trial were recorded direct to hard-disc using a condenser microphone. All stimulus presentation and response capture was performed using MatLab (MathWorks; Natick, MA) with the Psychophysics Toolbox.

##### Design and procedure

A 2 × 2 within-subject, repeated-measures design was employed, with modality (auditory, visual) and lexicality (word, nonword) as factors. Trials involved the serial presentation of five-item sequences. In both modalities, trial onset was cued by the appearance of the word “Ready” for 2 s, followed by a 1-s blank screen. Each item was presented for 250 ms. Items were separated by a 100-ms interval consisting of a blank screen (in the visual condition) or silence (in the auditory condition). Participants were instructed to begin spoken recall of the sequence at the offset of the final item (cued by the appearance of a centrally fixated question mark) and then press any key to end the trial. Explicit instructions were given to recall the items in the correct order, replacing any missing items with the word “Blank.” Trials were initiated automatically and were separated by a 1,500-ms interval.

Visual and auditory stimuli were presented separately. Each participant performed one test for each modality. Order of modality presentation was counterbalanced across participants. For each run, 20 five-item sequences of words and 20 five-item sequences of nonwords were selected randomly (without replacement) from the available 25 sequences in each set. Each test consisted of 40 unique sequences. Order histories of words and nonwords were (*n* − 1) counterbalanced within each test. An additional six practice trials were presented at the start of each test to familiarize participants with the procedure.

#### Results and discussion

Items were scored as correct only if both item identity and position were correct. The proportions of correctly recalled items were calculated for each of the four lexicality-by-modality combinations, at each serial position. In order to correct for possible violations of homogeneity of variance inherent in proportion data, scores were subjected to arcsine transformation prior to analysis. The transformed mean proportion correct scores were compared using within-subject, repeated-measures analyses of variance (ANOVAs), with modality, lexicality, and serial position as factors.

**Figure 1 fig1:**
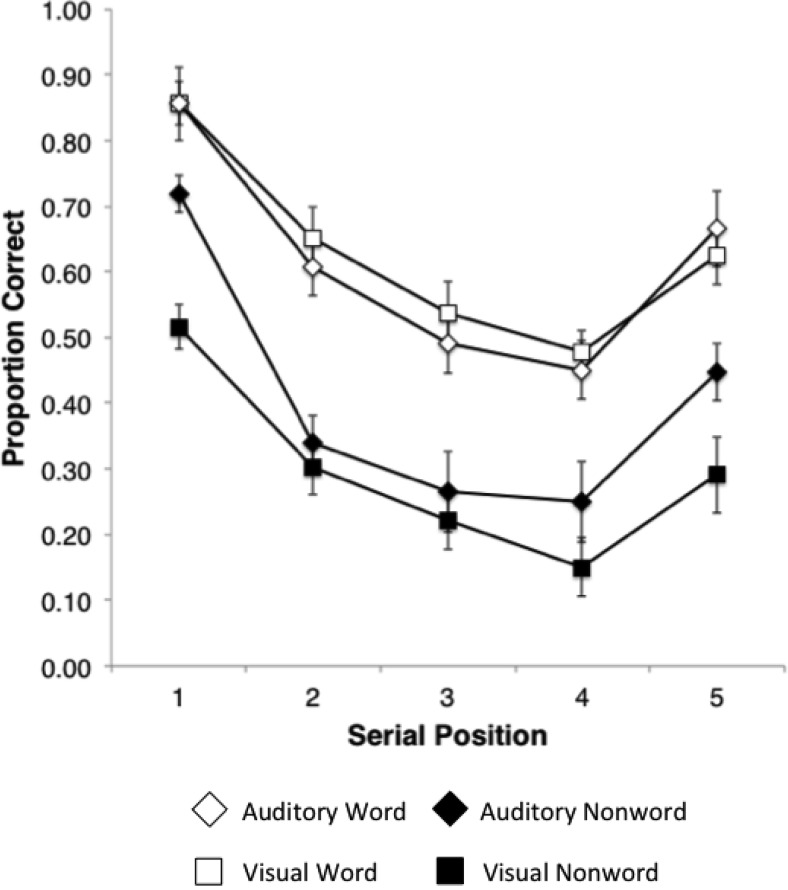
Mean serial position curves for recall of five-item sequences of words and nonwords in the auditory and visual modalities. Error bars denote standard error.

Serial position curves for the four combinations of lexicality and modality are shown in Figure 1. Visual inspection indicated superior recall of words compared with nonwords at all serial positions and in both modalities. Qualitatively similar patterns of results are seen for words in each modality; however, nonwords appear to exhibit superior recall in the auditory compared with the visual condition, with this advantage seen predominantly at initial and terminal sequence positions.[Fig-anchor fig1]

This impression was confirmed by a 2 × 2 × 5 repeated-measures ANOVA, with modality, lexicality, and serial position as within-subject factors. Main effects of lexicality, modality, and serial position were significant, *F*(1, 15) = 96.91, *p* < .001, η_p_^2^ = .87; *F*(1, 15) = 6.37, *p* = .024, η_p_^2^ = .30; and *F*(4, 60) = 57.73, *p* < .001, η_p_^2^ = .79, respectively. A significant two-way interaction was found between lexicality and modality *F*(1, 15) = 15.93, *p* = .001, η_p_^2^ = .52. All remaining interactions were not significant (*p* > .05).

**Figure 2 fig2:**
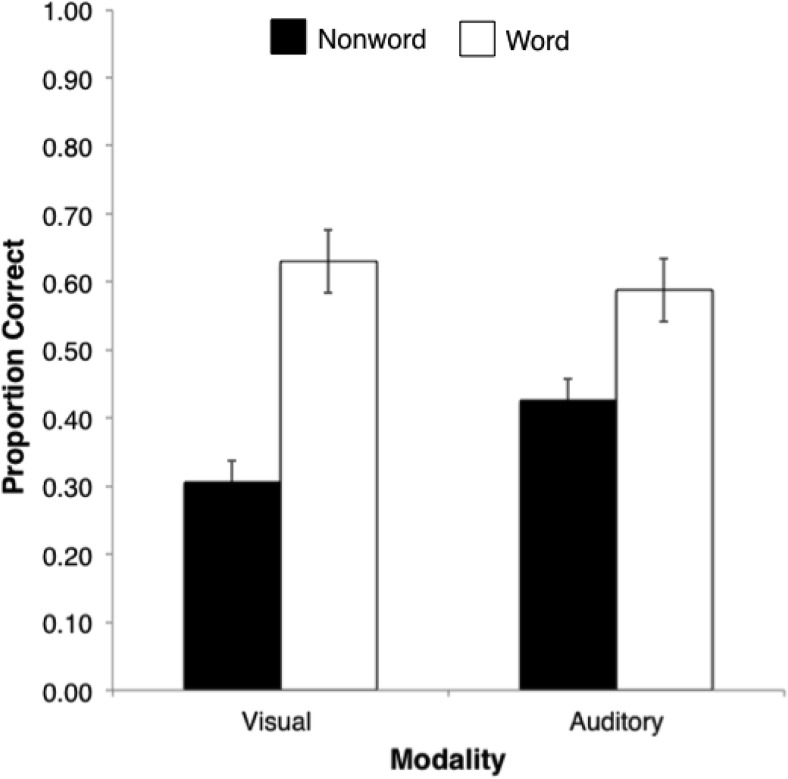
Mean serial recall performance (collapsed across serial position) in the visual (left) and auditory (right) modalities for five-item sequences of words and nonwords. Error bars denote standard error.

The prediction that the lexicality effect would be found for both visual and auditory serial recall was confirmed by separate 2 (lexicality) × 5 (serial position) repeated-measures ANOVAs in each modality. For visual presentation, the main effects of lexicality and serial position were significant, *F*(1, 15) = 112.21, *p* < .001, η_p_^2^ = .88, and *F*(4, 60) = 18.67, *p* < .001, η_p_^2^ = .55, respectively, as was also the case for auditory presentation, *F*(1, 15) = 40.63, *p* = .001; η_p_^2^ = .73, and *F*(4, 60) = 55.64, *p* = .001, η_p_^2^ = .79, respectively. The two-way interaction of lexicality and serial position was not significant for either modality (*p* > .05 in both cases). The effect of lexicality in each of the presentation modalities, collapsed across serial position is shown in Figure 2.[Fig-anchor fig2]

Further investigation was made into the significant interaction between modality and lexicality. The impression given by Figure 1 is that this might be driven by superior recall of nonwords for auditory over visual presentation at initial and terminal sequence positions in the nonword condition. This was confirmed by applying separate 2 (lexicality) × 2 (modality) repeated-measures ANOVAs at each serial position. The interaction of lexicality and modality was significant at Serial Positions 1, 4, and 5, *F*(1, 15) = 12.92, *p* = .003, η_p_^2^ = .43; *F*(1, 15) = 8.98, *p* = .009, η_p_^2^ = .38; and *F*(1, 15) = 5.57, *p* = .032, η_p_^2^ = .27, respectively, and not significant at Positions 2 and 3 (*p* > .05).

For serial recall, therefore, there were clear advantages for words over nonwords for both presentation modalities. Interestingly, the effect of lexicality was reduced somewhat for auditory, compared with visual, presentation, an effect arising primarily due to an advantage in the recall of nonwords presented auditorily compared with visually at the beginning and end of the sequence. We return to consider the implications of this aspect of the results in the light of the further findings of Experiment 1B, in which we tested auditory and visual serial recognition of the same type of sequence as that presented for serial recall in Experiment 1A.

### Experiment 1B

#### Method

##### Participants

Sixteen participants (14 women, mean age 21 years), none of whom had taken part in Experiment 1A, were recruited from the Cardiff University Human Participant Panel. Informed consent was obtained in accordance with Cardiff University School of Psychology ethics procedures.

##### Materials, design, and procedure

The verbal stimuli were the same as those used in Experiment 1A. Trials involved sequential presentation of a standard sequence, with the temporal parameters as used in Experiment 1A, followed by a 1,500-ms silent interval, followed by a test sequence either identical to the standard or differing by virtue of transposition of two adjacent items, not including the first or last items. Each of the remaining transpositions occurred an equal number of times across trials. Half of the trials contained identical standard and test sequences, with half being different. At the end of each trial, participants were probed to provide a *same/different* response via the computer keyboard. Conditions were arranged and counterbalanced in the same way as in Experiment 1A.

#### Results and discussion

**Figure 3 fig3:**
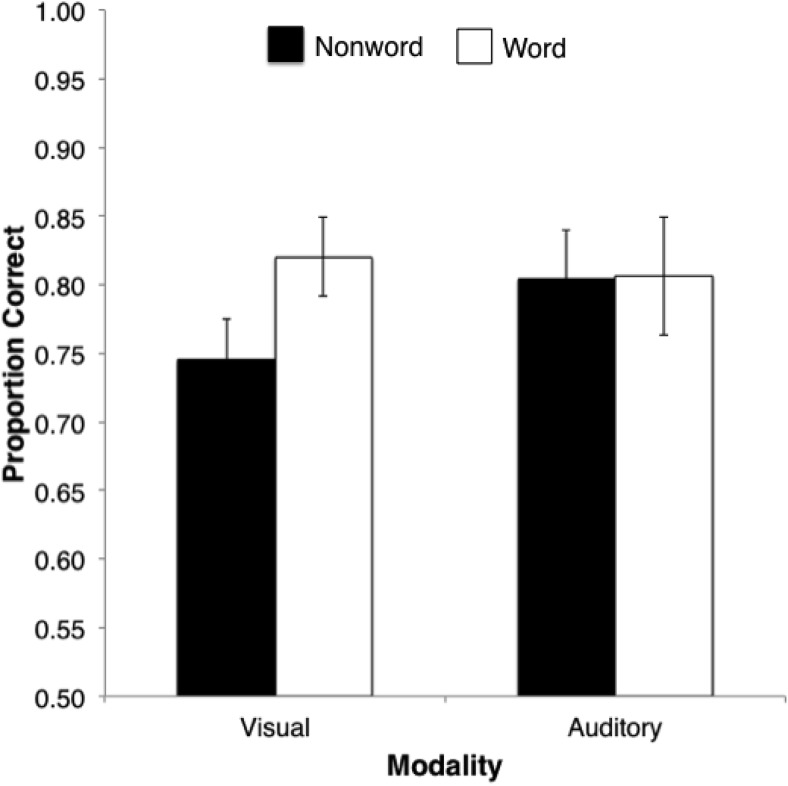
Mean serial recognition performance in visual and auditory modalities, for five-item sequences of words and nonwords. Error bars denote standard error.

In order to enable direct comparison with previous relevant findings in the literature, we first present analyses in terms of proportion correct, then present further analyses of accuracy broken down by trial type (*same/different*) as well as *d*′ (the latter typically not having been reported in the relevant literature on serial recognition discussed previously). We first calculated the proportion of correct responses for each participant, collapsing across *same* and *different* trials (see Figure 3). A within-subject, two-way, Modality × Lexicality, repeated-measures ANOVA revealed no main effects of either presentation modality or lexicality, *F*(1, 15) = 3.86, *p* = .54, η_p_^2^ = .03, and *F*(1, 15) = 3.80, *p* = .07, η_p_^2^ = .20, respectively. However, the interaction of lexicality and modality was significant, *F*(1, 15) = 7.17, *p* = .017, η_p_^2^ = .32. Pairwise comparisons (two-tailed) established that this interaction was due to an advantage for words over nonwords with visual serial recognition, but no lexicality effect for auditory presentation, *t*(15) = 3.43, *p* = .004, and *t*(15) = 0.28, *p* = .78, respectively.[Fig-anchor fig3]

Analyses of *d*′ differed from this pattern in that while there was a significant effect of lexicality, *F*(1, 15) = 5.32, *p* = .04, η_p_^2^ = .26, revealing better discrimination of word than nonword sequences, the effect of modality failed to reach significance, *F*(1, 15) = 3.71, *p* = .07, η_p_^2^ = .20, as did the interaction, *F*(1, 15) = 0,25, *p* = .88, η_p_^2^ = .02. The reason for the different pattern found here compared with that found with overall proportion correct can be seen in Table 1, which depicts proportion correct as function of trial type (*same/different*) as well as lexicality and modality. There is a clear tendency toward responding *same* rather than *different*, such that proportion correct is higher for *same* than for *different* trials in both auditory and visual modalities, *t*(15) = 4.97, *p* < .001, and *t*(15) = 4.51, *p* < .001, respectively. This brings correct responses for *same* trials therefore, close to ceiling in both modalities, potentially masking any effect of lexicality for those trials (a similar pattern is evident in the results of some conditions in Gathercole et al., 2001). However, equally clear is that in the below-ceiling accuracy in *different* trials, there is a lexicality advantage for visual presentation, *t*(15) = 2.86, *p* = .01, that is totally absent in the auditory modality, *t*(15) = 0.76, *p* = .46.[Table-anchor tbl1]

Taken together, the pattern of results in Experiments 1A and 1B broadly replicates those reported elsewhere on the influence of lexicality in different types of short-term memory task (e.g., Gathercole et al., 2001; Hulme et al., 1997; Roodenrys et al., 1993). Specifically, while such an influence is robustly and reliably demonstrable in serial recall, lexicality plays a smaller role in determining serial recognition performance. The first critical addition to this pattern from the current results, however, is that it is not the particular retrieval setting constituted by recognition that determines its relative immunity since matched presentation and retrieval conditions in auditory and visual serial recognition lead to a reliable advantage for words in the latter, and no such effect in the former modality. A second key novel demonstration here is that the role of lexicality in serial recall is also modulated by modality, with a smaller effect for auditory than visual serial recall, an effect principally confined to the beginning and end of the auditory sequences.

Taken together, these novel findings provide detailed support for our account of the role of lexicality in short-term memory, one in which the advantage accruing to lexically familiar material derives from the increased facility with which such material affords ready segmental recoding within the speech motor control processes that underpin performance in certain types of short-term memory task. By the same token, short-term memory tasks that do not rely upon such segmental recoding but may be accomplished on the basis of global, object-oriented perceptual processes are correspondingly immune to the effects of lexical familiarity. Before moving on in Experiment 2 to further elaboration of our account of how lexicality impacts on the recoding of verbal information, we first elaborate our account of auditory serial recognition, why it is potentially immune to any effect of lexicality, and how our account differs from that represented by already existing accounts.

As described previously, according to one account of the role of linguistic information in short-term memory performance (e.g., Baddeley, 2012; Gathercole et al., 2001), temporary phonological representations do not in themselves represent long-term linguistic knowledge, but they benefit from the existence of long-term linguistic representations that may be utilized to support the integrity of the corresponding short-term representations. Accounts that do not depend on separate short-term phonological representations, but view short-term memory as corresponding to the activated portion of long-term linguistic memory (e.g., Jefferies et al., 2005, 2006), account for the effects of linguistic familiarity by reference to the relatively robust activation that already-existing lexical–phonological representations receive by virtue of corresponding lexical and semantic levels of activation, as well as the relatively well-integrated lexical–phonological representations for established words over novel nonwords. The interaction between lexicality and retrieval conditions is correspondingly accounted for either by reference to involvement of processes of item redintegration, or the relative burden on item memory in different retrieval conditions. Both of these accounts share, therefore, an account couched in terms of the advantageous storage or activation of item-level phonological information for words compared with nonwords.

Our approach is fundamentally different to these, not only in its functional account of the key effects but also in its key explanatory concepts. We are proposing that auditory serial recognition is not best thought of as a task in which information about the phonological content of items and their ordering in a sequence needs to be retained from the standard to the test sequence. Rather, we see it as a task in which two integrated perceptual representations—what we refer to as *auditory* (analogously to visual) objects—are subject to a global-matching process, the success of which is determined not by the extent to which item information is either maintained or reproduced between standard and test sequences, but rather by the extent to which the presentation conditions are conducive to the formation of such auditory objects. Critically, as we discussed previously, the ability to do this is dissociable from the ability to access information about the individual items and their order within the sequence (see e.g., Warren, 1999). In this respect, acoustic (as opposed to phonological) content and timing are critical, so that those stimulus parameters that most readily yield well-integrated auditory objects, the comparison of which may enable global *same/different* judgments, provide those conditions in which the effects of lexicality are least likely to appear, since the perceptual processes afford a ready means of accomplishing the task without recourse to segmental speech motor processes.

From this point of view, it is worth noting that the presentation rate of the standard and test sequences in our Experiment 1B was faster than that used in, for example, the Gathercole et al. and Jefferies et al. studies described earlier. In both of those cases, significant effects of lexicality were found in serial recognition in some conditions and to varying degrees, although they were always small compared with the effects in serial recall. For example, Gathercole et al. reported effects sizes ranging from around η_p_^2^ = .15 to η_p_^2^ = .25, with larger effects typically being associated with longer sequence lengths. On the other hand, we found no effect whatsoever of lexicality for auditory serial recognition. The relevance of timing here is that the shorter the overall sequence duration, the greater the tendency for the constituents to be bound into a single auditory object, with slower rates of presentation effectively weakening the process of auditory object formation (e.g., Rowe & Cake, 1977; Warren, 1999). As such, our timing parameters (350 ms per item) were more likely (by design) to lead to coherent auditory object formation compared with those of Gathercole et al. (750 ms per item) and Jefferies et al. (1,000 ms per item) and therefore more likely to lead to conditions that readily afforded perceptual pattern matching as a basis for performing the task. In this way, the small but significant effect of lexicality in serial recognition in those previous demonstrations compared with the complete absence of an effect in our Experiment 1B is argued to be due to the more ready auditory object formation afforded by our fast rate of presentation.^3^ Of course, precisely the same timing parameters that rendered no effect of lexicality for auditory serial recognition did yield effects of lexicality in both serial recall and visual serial recognition, conditions where such auditory object formation could not provide a basis for task performance. This approach provides, then, a potentially coherent way to account for the variety of findings here and in the literature. Undoubtedly, further empirical investigation will determine the generality of an account of the influence of linguistic factors on short-term memory framed in terms of how perceptual-motor affordances interact with aspects of the type and form of verbal material presented to the participant (e.g., word/nonword, auditory/visual, fast/slow) and the particular type of task that the participant is required to carry out on that material (e.g., serial recognition/serial recall, discrimination/identification of syllabic order, discrimination/identification of subsyllabic segment order).

This emphasis on the role of auditory perceptual processing also provides a coherent account of the smaller effect of lexicality for auditory compared with visual serial recall. It has been demonstrated many times elsewhere (e.g., Jones et al., 2006; Maidment & Macken, 2012; Maidment et al., 2013; Nicholls & Jones, 2002) that the advantages seen in serial recall for auditory over visual lists (e.g., enhanced recency) derives from just such auditory perceptual processes. In this respect, processes of perceptual object formation privilege features at the boundaries of objects, given the key role played by boundary (or contour, as it is more usually referred to in relation to visual perception) information in constituting the object as an object in the first place (see e.g., Wagemans et al., 2012). The consequences of this can be seen in the greater accuracy with which information occupying the boundaries of auditory sequences may be identified, compared with information located “within” the perceptual object (e.g., Bregman & Rudnicky, 1975; Warren, Obusek, & Farmer, 1969). Therefore, what we see as the reduced effect of lexicality in auditory versus visual serial recall, one that is evident at the initial and terminal boundaries of the sequence, can be argued to be due to just the same object-oriented perceptual processes that are at play within the recognition setting, but in the case of recall, auditory perceptual processing cannot provide a full basis on which to accomplish the task, since an output version of the input sequence still needs to be segmentally encoded.

One of the implications of this account is that the functional character of a short-term memory task is determined less by its mnemonic characteristics—for example, in relation to number and type of items, retrieval conditions, and so on—but rather by the way in which the combined and distinct processes of perceptual organization and segmental motor coding may be brought to bear to accomplish the particular task goals with that particular material. Taking our current results along with previous demonstrations of the variable role of lexicality in different settings, we argue therefore that to the degree to which task conditions allow for perceptual processes involved with auditory object formation to occur and provide a basis for performance, then the effect of lexicality will be correspondingly small or absent. The other side of this argument is that the effect of lexicality emerges to the extent that the task involves the type of segmental recoding associated with speech motor processes, and we provide a further test of this in Experiment 2.

## Experiment 2

Thus far, we have argued that the effect of lexicality in short-term memory is a manifestation of the way in which performance is determined by the facility with which verbal material may be segmentally recoded and that therefore it will be absent to the extent that the task is accomplished without the engagement of such recoding processes. This, we have argued, is what accounts for its robust presence in serial recall and its smaller (or, as in the case of Experiment 1B, absent) effect in auditory serial recognition, since the latter may be accomplished on the basis of global perceptual processes that are distinct from such recoding (e.g., Burton et al., 2000; Macken et al., 2009; Warren, 1999; Warren et al., 1969). By the same token, impeding the deployment of such recoding processes during visual serial recognition should once again reduce or eliminate the influence of lexical status on performance. In Experiment 2, we tested this by comparing visual serial recognition performance under control conditions with conditions in which participants were required to engage in concurrent, task-irrelevant articulatory activity during the task. There are a number of lines of evidence that suggest that such articulatory suppression, rather than causing general impediment to the encoding of visual verbal material or indeed of phonological encoding more generally, is particularly damaging to segmental processing. For example, while articulatory suppression has a marked disruptive impact on visual–verbal rhyme judgments, homophone judgments are relatively immune to its effects (e.g., Besner, 1987; Besner, Davies, & Daniels, 1981; see also Tree, Longmore, & Besner, 2011). The key distinction between these two tasks is that while both tasks may be broadly thought of as “phonological,” rhyme judgments necessarily require segmentation of the verbal representation, in order to make a comparison between rime segments independent of syllable onsets, whereas no such segmentation is required to make a homophone judgment. The demonstration that articulatory suppression is especially disruptive of visual–verbal short-term memory tasks requiring the retention of the order of items (i.e., serial recall) compared with tasks that require only the short-term retention of item information (Macken & Jones, 1995) also points to its particular role in disrupting subvocally mediated segmental processing, rather than phonological encoding per se. As such, if the effect of lexicality found in visual–verbal serial recognition is located in processes involved with the segmental coding of verbal material, then it should be attenuated or abolished under articulatory suppression.

### Method

#### Participants

Thirteen participants (10 women, mean age 20 years) were recruited from the Cardiff University Human Participant Panel. Informed consent was obtained in accordance with Cardiff University School of Psychology ethics procedures.

#### Materials, design, and procedure

We manipulated lexicality (words/nonwords) and articulatory suppression (control/suppression) within subject using the same stimuli as used in Experiment 1B but at a slower rate of presentation in order to ensure that the additional burden due to articulatory suppression would not lead to floor effects in performance under those conditions. Each trial began with a fixation cross flashing at a rate of 2Hz for 3 s prior to the onset of the standard sequence. The five items in each sequence were presented at an onset-to-onset rate of 750 ms (on for 500 ms, off for 250 ms) and standard and test sequences were separated by a 1,500-ms interval. For articulatory suppression conditions, participants were instructed to begin quietly but overtly speaking “one, two, three, . . . ” repeatedly in time with the fixation cross and to continue doing so until the appearance of a question mark at the end of the test sequence prompted them to make the *same/different* judgment. For control trials, they were instructed to remain silent throughout. Supervised practice trials took place before commencement of the experimental trials to acquaint participants with the articulatory suppression requirements, and to promote compliance, we monitored the suppression throughout the experiment. Participants performed two tests (word, nonword) of 80 trials (40 *same*, 40 *different*). Each test was subdivided into four blocks—two each of control and articulatory suppression trials—in an alternating ABAB or BABA sequence. Thus, in each test, participants performed 20 trials each of *same* or *different* trials either with or without articulatory suppression. Test and block orders were fully counterbalanced across participants (note: the counterbalancing protocol was based on the 16 participants originally tested, three of whom had to be excluded due to chance performance; that is, in each case accuracy was not significantly above 50%).

### Results and Discussion

**Figure 4 fig4:**
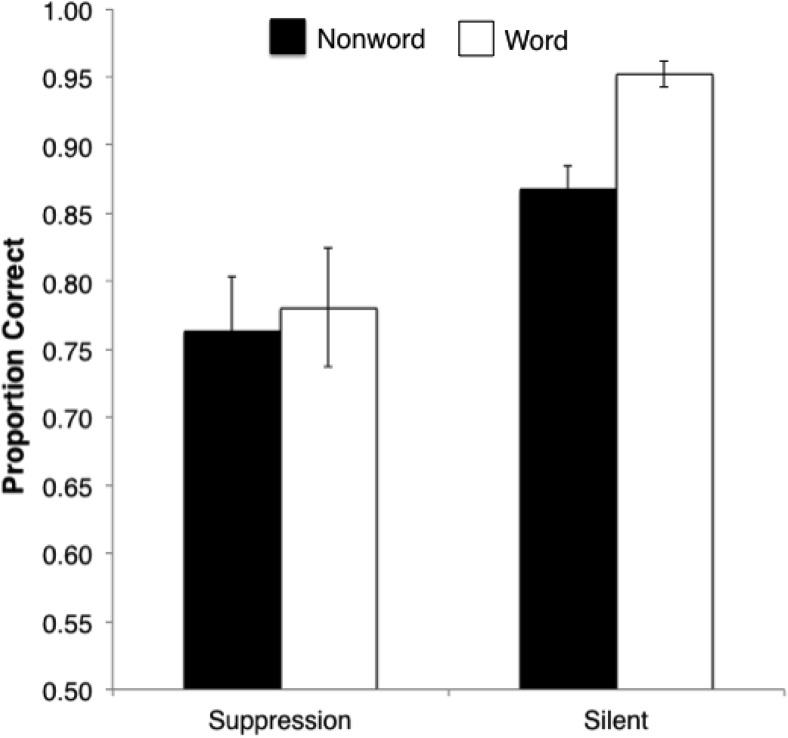
Mean proportion correct for serial recognition of five-item lists of words and nonwords, with and without articulatory suppression. Error bars denote standard error.

We first report analyses of overall proportion correct (see Figure 4), followed by proportion correct broken down by trial type (*same/different*) and *d*′. Mean proportion correct, collapsed across *same* and *different* trials, was calculated for each participant for each level of lexicality and suppression. In a 2 × 2 repeated-measures ANOVA, both main effects were significant: lexicality, *F*(1, 12) = 10.09, *p* = .008, η_p_^2^ = .46, and articulatory suppression, *F*(1, 12) = 19.76, *p* = .001, η_p_^2^ = .62. Critically, the interaction between suppression and lexicality was also significant, *F*(1, 12) = 5.13, *p* = .043, η_p_^2^ = .30, such that while word sequences were better recognized than nonwords under control conditions, *t*(12) = 6.54, *p* < .001, no such advantage occurred under articulatory suppression, *t*(12) = 0.63, *p* = .54. This interaction cannot be attributed to a floor effect in the suppression conditions as performance here was significantly above chance—words, *t*(12) = 5.32, *p* < .001, and nonwords, *t*(12), = 5.82, *p* < .001, respectively—and still at a level comparable to that found in Experiment 1B in which clear effects of lexicality were obtained.[Fig-anchor fig4]

This pattern was replicated in analysis of *d*′ (see Table 2), with main effects of both lexicality, *F*(1, 12) = 13.00, *p* = .004, η_p_^2^ = .52, and suppression, *F*(1, 12) = 12.67, *p* = .004, η_p_^2^ = .51, as well as a significant interaction, *F*(1, 12) = 5.32, *p* = .04, η_p_^2^ = .62. Simple effects indicated that this interaction was due to an advantage for words over nonwords under control conditions, *F*(1, 12) = 18.45, *p* = .001, η_p_^2^ = .61, that was abolished under articulatory suppression, *F*(1, 12) = 2.27, *p* = .16, η_p_^2^ = .16. While in Experiment 1B, analysis broken down by trial type (*same/different*) revealed a clear tendency to respond *same* rather than *different*, this pattern did not emerge in the corresponding analysis here, with no effect of trial type on proportion correct under control conditions, *t*(12) = 0.32, *p* = .75, or under suppression, *t*(12) = 1.43, *p* = .18. Furthermore, there was an advantage for words over nonwords in both *same* and *different* trials under control conditions, *t*(12) = 2.76, *p* = .02, and *t*(12) = 3.88, *p* = .002, respectively, which in both cases was eliminated under suppression, *t*(12) = 0.08, *p* = .94, and *t*(12) = 0.71, *p* = .48, respectively.[Table-anchor tbl2]

Here again, we have clear evidence that it is not the retrieval conditions per se that determine whether lexicality influences short-term sequence memory, but rather it is determined by the extent to which performance is based on segmental recoding processes associated with speech control mechanisms. When such processes are engaged, as in the case of serial recall, regardless of modality of presentation (notwithstanding the potential contribution of auditory perceptual processes with auditory presentation as discussed previously), and in serial recognition when the material is presented in visual form, then we see robust effects of lexical status of the material. When the role of such processes is mitigated, either by virtue of the availability of perceptual affordances that may be utilized to perform the task or by direct impediment to the deployment of them as in this experiment, then concomitantly the effect of lexical status recedes.

## General Discussion

These findings are problematic for traditional accounts of the role of long-term linguistic knowledge in short-term verbal memory performance. One approach invokes a distinct short-term memory system in which temporary phonological representations do not themselves represent long-term linguistic knowledge. However, such knowledge has a bearing on performance via a redintegrative process that supports retrieval of the degraded short-term representations. From this view, the smaller effect of lexicality in serial recognition compared with recall is due to re-presentation of all the study information in the test cue, thereby attenuating or eliminating the role of redintegration. Alternatively, the view that sees short-term memory as the temporary activation of long-term linguistic representations accounts for the reduced influence of lexicality on serial recognition by reference to reduced task sensitivity by virtue of the reduced burden on item identity, the latter being the locus of the linguistic influence on short-term memory performance. Each of these accounts would predict attenuated or absent effects of lexicality in both auditory and visual serial recognition, and we show this prediction to be incorrect. Further, the effect of lexicality present in visual serial recognition is eliminated under conditions of articulatory suppression. This suggests that the lexicality effect emerges in visual serial recognition as a consequence of the requirement in that task to engage speech control mechanisms to segmentally recode the visual–verbal information. When the deployment of such processes is impeded, the lexicality effect is absent. In turn, this points to an explanation of the diminished role of lexicality in auditory serial recognition not as a consequence of retrieval conditions per se, but rather as a consequence of the task-specific engagement in such a setting of perceptual mechanisms that afford sequence matching independent of the segmental processes accomplished via speech motor mechanisms.

So, in distinction to both these traditional views of short-term memory, we instead propose that short-term memory performance represents the opportunistic and task-specific deployment of processes that are not best conceived of as serving memory per se, but rather as being involved in the auditory perceptual organization and motor encoding of sequences of verbal material. As such, we propose that the influence of lexicality in short-term memory is amenable to an account in terms of the affordances of different types of verbal material and different modalities of presentation. One aspect of this is the way in which the auditory modality affords sequence matching without the necessary engagement of segmental speech control processes. This affordance we have elaborated in some detail in the discussion of Experiments 1A and 1B.

The other way in which the concept of affordance provides a powerful way to conceptualize short-term memory performance is in relation to how familiarity with a set of verbal material enhances the readiness and economy with which that material may be encoded into a sequence of articulatory gestures. The frequency with which lexical and supralexical components occur within normal communicative speech impacts on the degree to which sublexical and subsyllabic elements are fully expressed in articulatory execution. Typically, lenition involves reduction of consonant sounds (e.g., the flapping of a terminal /t/) but may also lead to the reduction of larger sublexical segments. For example, The schwa-plus-/r/ segments in the low-frequency *artillery* are fully articulated, reducing to a syllabic /r/ in the medium frequency *memory*, with the schwa segment disappearing completely in the high-frequency *every* (Hooper, 1976; see also Bauer, 2008). Such a process can readily account for the finding that articulatory duration tends to reduce as a function of lexical frequency, especially when sequences, rather than single isolated instances, of lexical items are produced (Wright, 1979). Indeed the priority of the extended utterance over its elements can be seen even to the extent of the complete elimination of lexical-semantic constituents from an utterance, as in the frequency-driven diachronic transition from *do not know* to *don’t know* to *dunno* (see Bybee, 2010).

In such a way, lexically familiar material may be seen to afford more ready encoding and rehearsal than less familiar, or unfamiliar, lexical material. Such a claim might at first appear to be at odds with a number of demonstrations in the literature that appear to rule out a role for articulatory factors in giving rise to the impact of linguistic familiarity on short-term memory performance (e.g., Gregg, Friedman, & Smith, 1989; Hulme, Roodenrys, Brown, & Mercer, 1995, Hulme, Roodenrys, Schweickert, et al., 1997; Stuart & Hulme, 2000; Thorn et al., 2002). Typically, such claims are based on two types of finding: one, that linguistic familiarity retains an influence even when articulatory duration is controlled, either statistically, or by selecting materials from familiar and unfamiliar classes that are matched on articulatory duration; and two, that linguistic factors retain an influence on performance under conditions of articulatory suppression.

With regard to each of these, there are a number of critical caveats that need to be borne in mind in evaluating the implications of such findings for an understanding of the impact of linguistic familiarity on short-term memory. In relation to the question of the control of articulatory duration, the precise way in which it is measured turns out to be critical. Typically, the articulatory duration for a set of material has been established by requiring participants to utter aloud repeated single or pairs of items. However, as we have already noted, the influence of familiarity on articulatory fluency is increasingly evident in extended utterances. Not only is this likely due to the increasing manifestation of lenition in such circumstances, but there is also a specific influence of familiarization on fluency of co-articulatory transitions between lexical items (Woodward et al., 2008). This means that utterances involving only singles or pairs are least likely to show evidence of an effect of linguistic familiarity on duration. Indeed, sets of high- and low-frequency words that have previously been used to argue against the articulatory basis of frequency effect in serial recall (e.g., Hulme *et al.* 1997) turn out, when duration for articulation of six-item sequences rather than singles or pairs is measured, to show a substantially reduced duration for high- compared with low-frequency words (Woodward et al., 2008).

A perhaps more fundamental question is whether articulatory duration in itself is actually an important determinant of short-term memory performance. It is in relation to the maintenance of temporally limited phonological representations that the role of articulatory duration pertains: the fewer items that may be rehearsed in a given unit of time, the poorer their retention will be (e.g., Baddeley, 2012; Baddeley, Lewis, & Vallar, 1984). However, effects previously attributed to articulatory duration turn out instead to be mediated by articulatory complexity (e.g., Caplan, Rochon, & Waters, 1992; Service, 1998). Evidence such as this has led to the argument that temporal decay cannot explain limitations in short-term memory performance (e.g., Lewandowsky & Oberauer, 2009; Nairne, 2002), and therefore, the predictive value of duration in the first place comes under question. Therefore, even in sets of high- and low-familiarity verbal materials that are putatively matched on articulatory duration, by whatever measure, the question remains as to whether complexity, as a factor imperfectly related to duration, is also matched. The idea that complexity, rather than speed of processing, plays a role in short-term verbal memory lends itself readily to an account of performance in terms of the affordances within the verbal material for facile articulatory coding.

This leaves the question of the survival of effects of linguistic familiarity under conditions of articulatory suppression (e.g., Gregg et al., 1989), and here too, critical aspects of methodology warrant a reappraisal of the implications. The mechanism whereby articulatory suppression is typically held to disrupt short-term memory is by impeding subvocal processes involved in the orthography-to-phonology conversion that yield phonological representations or in the processes of subvocal rehearsal necessary to prevent decay in the activation of those representations (e.g., Baddeley & Hitch, 1974). Its role in maintaining activation is, in broad terms, common regardless of whether short-term memory is regarded as a separate system or as an activated portion of long-term memory (e.g., Baddeley, 2012; Cowan, 1995; Ruchkin et al., 2004). One of the implications of this is that mere task-irrelevant engagement of articulatory processes is generally deemed to be sufficient for suppression to have its effect on processing (e.g., Baddeley, 1990). However, it turns out that different types of suppression have functionally and quantitatively different effects on performance. So, for example, suppression involving repetition of a single letter sound (e.g., “*A, A, A, A, A* . . .”) is less disruptive of serial recall than suppression involving an overlearned sequence of letter sounds (e.g., “*A, B, C, A, B, C* . . .”). Furthermore, overt suppression adds an additional independent degree of disruption compared with silent “inner” suppression, even though the latter still exhibits the influence of changing versus repeated suppression (Macken & Jones, 1995). Clearly, then, a more fine-grained analysis of the impact of task-irrelevant articulatory activity on short-term memory performance is required, beyond one that merely attributes its general impediment to subvocal rehearsal.

This in turn raises the possibility that different implementations of articulatory suppression impacts on different levels of the specification of subvocal speech control mechanisms subsuming rehearsal of a verbal sequence. Such a possibility is also pointed to by the distinction between the impairments found in speech apraxia, where processes of speech planning are impaired, and disarthria, where control of the muscles of the vocal tract is impaired. While patients suffering from the latter exhibit the hallmarks of typical verbal serial recall, such as effects of phonological similarity and word-length, the former do not (e.g., Rochon, Caplan, & Waters, 1990; Waters, Rochon, & Caplan, 1992). Given this, we would suggest that the interaction between linguistic familiarity and articulatory suppression warrants a closer look. However, for present purposes, we can refer to the novel demonstration here that a robust effect of lexical status on visual–verbal serial recognition is completely abolished by concurrent overt uttering of a task-irrelevant sequence.

In our focus on concepts of affordance and perceptual-motor processing, we are proposing a general framework for examining short-term memory that deviates from many of the assumptions of more traditional cognitivist accounts. At its most basic, the theorizing of short-term verbal memory posits systems for the maintenance or activation of representations whose content is phonological. The lineage here leads directly to Chomskyan psycholinguistics (e.g., Chomsky & Halle, 1968) in which the elements of verbal utterances are abstract phonological segments that may be lawfully assembled to provide control programs for the articulatory apparatus in order to produce the purported variety of speech acts. The genesis of the cognitive psychology of short-term memory established, so it seemed, the appropriateness of this abstract phonological level of representation as the essential currency of verbal short-term memory (e.g., Baddeley, 1966; Conrad & Hull, 1964). This was because the short-term memory deficit found with a sequence of verbal tokens corresponding to similar phonological representations compared with those corresponding to dissimilar ones occurred regardless of whether those tokens were presented in auditory or written form. In this way, a level of representation transcending modality was implicated, and nearly 50 years of research has almost universally conformed to this view. However, as we alluded to earlier, a considerable amount of recent evidence has shown that this classic phonological similarity effect points not to a shared level of phonological representation regardless of modality of input, but rather to distinct and combined roles for articulatory and auditory similarity in reducing performance. Aspects of performance previously attributed to interactions among modality-independent phonological representations can be shown to be fully accounted for by the distinct and combined operation of auditory perceptual and articulatory control processes (Jones et al., 2004, 2006; Maidment & Macken, 2012).

Not only has the operation of so-called phonological similarity been located within modality-dependent perceptual-motor processes but so too has a broad range of other canonical aspects of verbal short-term memory performance, including the effect of task-irrelevant background sound on performance (e.g., Jones & Macken, 1993; Macken et al., 2009), the effect of voice change within a to-be-remembered auditory sequence (Hughes, Marsh, & Jones, 2009); the effect of modality of presentation on recall, and the effect of an end-of list suffix on auditory recency (Nicholls & Jones, 2002), the effect of articulatory suppression and word length (Macken & Jones, 1995; Tremblay, Macken, & Jones, 2000) and the effect of articulatory complexity (Murray & Jones, 2002). This approach not only eschews the basic assumptions of traditional short-term memory theorizing but in so doing also abjures the Chomskyan heritage underpinning it. In this way, our framework also brings the study of short-term memory more in line with those contemporary accounts that have undermined the role of phonology more generally as a unique form of representation underpinning human verbal behavior and have instead sought to locate accounts of verbal behavior within general frameworks for explaining perception and action (see e.g., Bybee, 2010; Goldstein, Pouplier, Chen, Salzman, & Byrd, 2007; Hickok & Poeppel, 2004; Port, 2010; Port & Leary 2005). What we have shown in the experiments here is that the lexicality effect, an effect typically attributed either to the retrieval of bespoke temporary phonological representations or to the temporary activation of established ones, can in fact be accounted for within a general framework of affordances within perceptual-motor processing. In so doing, the results here lend further weight to an account of short-term memory that, rather than viewing perceptual and motor processes as merely placing input and output constraints on a separate short-term memory system, are in fact the very embodiment of that system.

## Figures and Tables

**Table 1 tbl1:** Mean (and SD) Values for d′ Along With Proportion Correct for Same and Different Trials as a Function of Lexicality and Modality in Experiment 1B

Variable	*d*′		Proportion correct			
			Same		Different	
	Word	Nonword	Word	Nonword	Word	Nonword
Auditory	3.04 (2.47)	2.44 (2.27)	0.92 (0.13)	0.88 (0.16)	0.69 (0.23)	0.72 (0.17)
Visual	2.04 (1.19)	1.53 (1.18)	0.87 (0.14)	0.84 (0.15)	0.76 (0.15)	0.64 (0.14)

**Table 2 tbl2:** Mean (and SD) Values for d′ Along With Proportion Correct for Same and Different Trials as a Function of Lexicality and Modality in Experiment 2

Variable	*d*′		Proportion correct			
			Same		Different	
	Word	Nonword	Word	Nonword	Word	Nonword
Suppression	2.49 (2.03)	1.88 (1.48)	0.82 (0.17)	0.81 (0.13)	0.75 (0.19)	0.71 (0.17)
Control	4.58 (1.68)	2.74 (1.11)	0.92 (0.09)	0.87 (0.09)	0.96 (0.04)	0.86 (0.08)

## Footnotes

1 The influence of linguistic knowledge is also manifest in individuals’ superior performance for high- versus low-frequency words, nonwords that conform to the phonotactic regularities of the known language versus those that do not, and, in bilingual “rememberers,” superior recall for first- versus second-language material.

2 This is not to say that serial recall does not also manifest the effects of auditory perceptual processing; recent analyses have shown that the differences between auditory- and visual-verbal serial recall can be accounted for in terms of the way in which such auditory perceptual processes play out as a function of the particular parameters of the recall task (e.g., Maidment & Macken, 2012; Maidment et al., 2013). However, given the requirement to reproduce a sequence, serial recall also necessarily requires the engagement of segmental processes in order to recode the presented sequence, in whatever modality, into an output sequence. We discuss this further when considering the results of Experiments 1A and 1B.

3 It might be argued that a redintegration account would make a similar prediction about the effect of timing, since slower presentation rates would be argued to lead to greater item degradation, in turn increasing the role that redintegration may play in performance. However, an account framed in terms of such degradation and retrieval processes would have to make broadly the same predictions about visual and auditory serial recognition, predictions that, as we have shown, are not borne out by the evidence.
